# Association between low-density lipoprotein cholesterol levels and serum uric acid to serum creatinine ratio in Chinese male gout patients

**DOI:** 10.1038/s41598-024-59992-7

**Published:** 2024-05-02

**Authors:** Qian Shao, Jingwei Chi, Kui Che, Yue Zhou, Yajing Huang, Yunyang Wang, Yu Xue, Yangang Wang

**Affiliations:** 1 Department of Endocrinology and Metabolism, the Affiliated Hospital of Medical College Qingdao University, Qingdao, China; 2 Department of Endocrinology, the Affiliated Hospital of Jining Medical University, Jining Medical University, Jining, China; 3 Qingdao Key Laboratory of Thyroid Diseases, the Affiliated Hospital of Medical College Qingdao University, Qingdao, China; 4 Department of Endocrinology and Metabolism, Huashan Hospital, Shanghai Medical School, Fudan University, Shanghai, China

**Keywords:** Cardiology, Diseases, Endocrinology

## Abstract

This study aimed to evaluate the association between low-density lipoprotein cholesterol (LDL-C) and serum uric acid to serum creatinine (SUA/SCr) ratio in male gout patients at different BMIs. This real-world study included 956 male gout patients aged 18–83 years. We retrospectively analyzed the medical records of Chinese male gout patients from 2017 to 2019. The correlation between LDL-C and SUA/SCr was tested after adjusting for confounding factors. We found a nonlinear relationship between LDL-C and SUA/SCr in the whole study population. Stratification analysis showed that there was actually a nonlinear relationship between LDL-C and SUA/SCr in men with a BMI of 24–28, the inflection point of LDL-C was 1.8 mmol/L, when LDL-C was greater than 1.8 mmol/L, there was a positive correlation between LDL-C levels and SUA/SCr (β = 0.67, 95% CI 0.35–0.98, *P* < 0.001). Moreover, LDL-C showed a significant positive correlation with SUA/SCr with a BMI of 28 or greater (β = 0.30, 95% CI 0.05–0.55, *P* = 0.019). However, no association was found between LDL-C and SUA/SCr with a BMI of less than 24 (β = 0.42, 95% CI − 0.03–0.86, *P* = 0.070). LDL-C levels were associated with SUA/SCr in Chinese male gout patients, but this correlation appeared inconsistent among different BMIs. Our findings suggest that LDL-C levels may be more noteworthy in overweight and/or obese male gout patients.

## Introduction

Gout is an inflammatory arthritis caused by the formation and deposition of monosodium urate monohydrate (MSU) in joints and other tissues. An elevated serum uric acid (SUA) level is the main causative factor for gout development. Gout occurs more frequently in men than in women, and studies from Asia have shown that the sex ratio of males to females is approximately 8:1^1^, which is significantly higher than that in Europe and North America^2–4^. As is well known, dyslipidaemia and obesity are relatively common in patients with gout. The “American Society for Preventive Cardiology (ASPC)Top Ten CVD Risk Factors” has listed ten significant risk factors for cardiovascular disease (CVD) since 2020, among these risk factors include obesity and dyslipidaemia^5^. Although many studies have linked hyperuricaemia with cardiovascular disease (CVD)^6,7^, whether high SUA levels or gout plays a causal role in the pathological mechanism in these diseases is controversial^8^.

As a component of the metabolic syndrome, dyslipidaemia has been reported to account for approximately 50–70% of gout patients in different countries^9–12^. Studies have demonstrated that SUA is associated with lipid profiles, but the complex relationship between them has not been fully elucidated. Low-density lipoprotein cholesterol (LDL-C), which accumulates and is oxidized in the subendothelial space, is an important factor in atherosclerotic plaque formation^13^. However, there are limited data on the relationship between LDL-C and SUA in gout patients. Furthermore, it has been shown that body mass index (BMI) is a significant risk factor for hyperuricaemia^14^. In men, the risk of gout increased significantly when the BMI was 25 or greater^15^. Moreover, studies have suggested that a higher BMI correlates with increased levels of triglycerides (TGs), total cholesterol (TC), and LDL-C, and decreased HDL-C^16^. Therefore, we speculate whether there is some association between LDL-C, SUA and BMI. Considering that SUA levels are often affected by abnormal renal function, renal function-normalized SUA (serum uric acid to serum creatinine ratio, SUA/SCr) has emerged as a biomarker and more accurately reflects net SUA levels^17,18^. Previous studies have found that higher SUA/SCr was significantly associated with metabolic syndrome and its components in type 2 diabetes patients and middle-aged and elderly people^19,20^, and similar findings have recently been reported in overweight and obese individuals^21^. However, to our knowledge, there is no literature reporting the relationship between LDL-C levels and SUA/SCr with different BMIs in gout patients.

In clinical practice, atorvastatin is often used in combination with uric acid-lowering drugs in gout patients with hypercholesterolemia for its additional uric acid-lowering effects^22,23^. Although the exact mechanism remains unclear, it appears that the decreased levels of SUA is to some extent related to the reduction of cholesterol. Therefore, in our current study, we retrospectively collected the data of male gout patients in China to explore the relationship between LDL-C and SUA/SCr at different BMIs.

## Methods

### Study population

We performed a retrospective cross-sectional study using medical records from the Department of Endocrinology at the Affiliated Hospital of the Medical College Qing Dao University (Qing Dao, China). The data of 956 male gout patients from 2017 to 2019 were included. The diagnostic criteria for gout were based on the gout classification criteria of the 2015 ACR/EULAR^24^. All included participants were hospitalized patients. We excluded patients who met any of the following criteria: (I) aged < 18 years; (II) female patients; (III) patients with lipid-lowering therapy; and (IV) history of diabetes, myelodysplastic disease, severe liver disease, severe heart or renal diseases, mental illness and malignant tumour. The Affiliated Hospital of Medical College Qingdao University approved this retrospective study (QYFY WZLL 27310). All methods explained here were performed in accordance with relevant guidelines and regulations. All participants gave written informed consent for the study.

### Data collection

Demographic and clinical variables included age, height, weight, duration of gout, history of smoking and drinking, systolic blood pressure (SBP) and diastolic blood pressure (DBP). Height, weight and blood pressure were measured using standard methods. We defined hypertension as being on antihypertensive therapy or a systolic pressure above 140 mmHg and/or a diastolic blood pressure above 90 mmHg. Body mass index (BMI) was calculated using the weight (kg)/height squared (m^2^) formula. The determination of overweight or obesity was made according to the following: overweight, 24.0 kg/m^2^ ≤ BMI < 28.0 kg/m^2^. obesity, BMI ≥ 28.0 kg/m^2^. Blood samples were collected in the early morning following at least an 8-h overnight fast. We used high-performance liquid chromatography (Bio-Rad Variant II HbA1c analyser; Bio-Rad, Hercules, CA, USA) to detect HbA1c. The DIMENSION LXR autoanalyzer (SIEMENS, Munich, Germany) was used to measure SUA, Renal function-normalized SUA were calculated using SUA/Scr, total cholesterol (TC), high-density lipoprotein cholesterol (HDL-C), low-density lipoprotein cholesterol (LDL-C), triglyceride (TG), fasting plasma glucose (FPG) and alanine aminotransferase (ALT). Serum creatinine was tested by Beckman Coulter AU 680 (Krefeld, Germany). The Chronic Kidney Disease Epidemiology Collaboration (CKD-EPI) formula was used to calculate the estimated glomerular filtration rate (eGFR)^25^. Dyslipidaemia was defined as satisfying one of the following conditions^26^: (1) TG ≥ 1.69 mmol/L (150 mg/dl), (2) TC ≥ 5.18 mmol/L (200 mg/dl), (3) LDL-C ≥ 3.367 mmol/L (130 mg/dl), and (4) HDL-C < 1.036 mmol/L (40 mg/dl).

### Statistical analysis

All statistical analyses were performed using R3.4.3 (http://www.r-project.org) and Empower Stats (http://www.empowerstats.com, X&Y Solutions, Inc. Boston MA). Normal distribution parameters are shown as the mean ± standard deviation (SD), nonnormal distribution parameters were expressed as the median (interquartile range), and categorical data are expressed as numbers and percentages (*n*, %). For the comparison between three groups, one-way analysis of variance (ANOVA) and Kruskal–Wallis tests were conducted for normal and nonnormal distribution parameters, respectively, and Chi square tests were used for categorical variables. We used a univariate analysis model to determine the association of LDL-C and other variables with SUA/SCr. Then, smooth curve fittings were performed to evaluate the relationship between LDL-C levels and SUA/SCr in the whole population and stratified by BMI after adjusting for confounders. Finally, we applied multiple linear regression models and piecewise linear regression models to further explore the relationship between LDL-C levels and SUA/SCr, and a *P* value < 0.05 was considered statistically significant (two-tailed).

## Results

### Clinical and laboratory characteristics of the subjects

The clinical and laboratory characteristics of the study subjects are presented in Table 1. This study enrolled 956 male gout patients aged 18 to 83 years. The proportion of gout patients with dyslipidaemia was approximately 72.60%. The mean LDL-C and SUA levels were 2.91 ± 0.76 mmol/L and 8.15 ± 2.13 mg/dl, respectively. Compared with the normal weight group, the overweight and obese groups had younger age, shorter disease duration, higher SBP, DBP, ALT and SUA/SCr, and higher proportion of dyslipidaemia. However, there was no significant difference among the three groups in the proportion of receiving urate-lowering therapies.

**Table 1:** Clinical and biochemical characteristics of the participants.

Variables	All	BMI < 24	24 ≤ BMI < 28	BMI ≥ 28	*P* value
Number	956	119	421	416	–
Age (years)	46.46 ± 14.53	54.71 ± 15.59	48.80 ± 13.34	41.73 ± 13.76	< 0.001
BMI (kg/m^2^)	27.76 ± 3.89	21.78 ± 1.75	26.12 ± 1.16	31.15 ± 2.85	< 0.001
SBP (mmHg)	135.00(125.00–146.00)	131.00(117.50–146.00)	132.00(123.00–145.00)	138.00(129.75–147.00)	< 0.001
DBP (mmHg)	84.79 ± 11.83	79.30 ± 11.88	83.66 ± 11.24	87.50 ± 11.70	< 0.001
Duration of disease (years)	7.00 (3.00–10.00)	9.00 (3.50–17.00)	8.00 (4.00–13.00)	6.00 (3.00–10.00)	< 0.001
HbA1C (%)	5.60 (5.30–5.90)	5.60 (5.30–6.00)	5.60 (5.30–5.90)	5.60 (5.40–5.90)	0.564
FPG (mmol/L)	4.90 (4.48–5.43)	4.85 (4.35–5.51)	4.88 (4.48–5.38)	4.95 (4.54–5.46)	0.419
SUA (mg/dl)	8.15 ± 2.13	8.06 ± 2.05	7.81 ± 2.09	8.54 ± 2.13	< 0.001
TG (mmol/L)	1.55 (1.13–2.32)	1.19 (0.88–1.67)	1.46 (1.09–2.23)	1.76 (1.28–2.64)	< 0.001
TC (mmol/L)	4.81 ± 0.99	4.58 ± 0.98	4.77 ± 0.99	4.92 ± 0.99	< 0.001
LDL-C (mmol/L)	2.91 ± 0.76	2.71 ± 0.80	2.91 ± 0.72	2.97 ± 0.78	< 0.001
HDL-C (mmol/L)	1.08 (0.95–1.26)	1.17 (0.97–1.40)	1.06 (0.94–1.25)	1.08 (0.95–1.24)	0.01
ALT (U/L)	30.00 (19.00–53.00)	21.00 (14.00–39.00)	27.00 (18.00–44.00)	35.00 (23.00–65.00)	< 0.001
SCr (mg/dl)	0.97 (0.78–1.14)	1.00 (0.86–1.24)	0.98 (0.78–1.15)	0.95 (0.77–1.12)	0.048
eGFR (mL/min/1.73 m^2^)	92.57 ± 28.40	80.91 ± 29.85	90.65 ± 27.55	97.86 ± 27.63	< 0.001
SUA/SCr	5.87 ± 2.20	5.30 ± 2.04	5.65 ± 2.23	6.26 ± 2.14	< 0.001
Hypertension, n(%)	458(47.91%)	54 (45.38%)	193 (45.84%)	211 (50.72%)	0.310
Dyslipidaemia, n(%)	694(72.60%)	66 (55.46%)	311 (73.87)	317 (76.20)	< 0.001
Smoking, n(%)	484 (50.63%)	73 (61.86%)	215 (51.19%)	196 (47.23%)	0.019
Drinking, n(%)	608 (63.60%)	83 (69.75%)	278 (66.83%)	247 (59.52%)	0.034
Treatment, n (%)					0.730
UA lowering therapy	669 (69.98%)	83 (69.75%)	300 (71.26%)	286 (68.75%)	
No medication	287 (30.02%)	36 (30.25%)	121 (28.74%)	130 (31.25%)	

BMI, body mass index; DBP, diastolic blood pressure; SBP, systolic blood pressure; FPG: fasting plasma glucose; SUA, serum uric acid; TG, triglyceride; TC, total cholesterol; LDL-C, low density lipoprotein cholesterol; HDL-C, high density lipoprotein cholesterol; ALT, alanine aminotransferase; SCr, serum creatinine; eGFR, estimated glomerular filtration rate, SUA/SCr, serum uric acid to serum creatinine ratio. Normally distributed data were shown as mean ± standard deviation, and non-normally distributed data were exhibited as median (interquartile range). The categorical data were shown as number (percentage). *P* < 0.05 is considered to be statistically significant.

### Associations between study parameters and SUA/SCr

As shown in Table 2, univariate analysis revealed the association between the study parameters and SUA/SCr. In the overall male gout patients, we noticed a significant positive relationship between LDL-C levels and SUA/SCr (β = 0.55, 95% CI 0.37–0.73, *P* < 0.001); meanwhile, other variables that significantly correlated with SUA/SCr were age, BMI, FPG, TG, TC, eGFR, ALT and hypertension. In addition, when grouped according to BMI, LDL-C, TG, and eGFR were positively correlated with SUA/SCr, while hypertension was negatively correlated with SUA/SCr, regardless of the normal weight group, overweight group, or obese group (*P* < 0.05). However, no association was found between DBP, SBP, HDL-C, smoking, drinking, treatment and SUA/SCr in the overall population and subgroups.

**Table 2:** Association between SUA/SCr and different variables conducted by univariate analysis.

Variables	All (n = 956)		BMI < 24 (n = 119)		24 ≤ BMI < 28 (n = 421)		BMI ≥ 28 (n = 416)	
	β(95%CI)	*P* value	β(95%CI)	*P* value	β(95%CI)	*P* value	β(95%CI)	*P* value
Age (years)	− 0.05 (− 0.06, − 0.04)	< 0.001	− 0.02 (− 0.04, 0.01)	0.128	− 0.05 (− 0.07, − 0.04)	< 0.001	− 0.05 (− 0.07, − 0.04)	< 0.001
BMI (kg/m^2^)	0.09 (0.02, 0.15)	0.009	0.00 (− 0.21, 0.21)	0.994	0.08 (− 0.10, 0.27)	0.386	0.10 (0.02, 0.17)	0.009
SBP (mmHg)	− 0.00 (− 0.01, 0.01)	0.569	0.00 (− 0.01, 0.02)	0.632	− 0.01 (− 0.02, 0.00)	0.099	0.00 (− 0.01, 0.02)	0.544
DBP (mmHg)	− 0.00 (− 0.01, 0.01)	0.708	0.03 (− 0.00, 0.06)	0.079	− 0.01 (− 0.03, 0.00)	0.135	0.00 (− 0.02, 0.02)	0.976
Duration of disease (years)	− 0.01 (− 0.03, 0.01)	0.276	0.03 (− 0.01, 0.08)	0.133	− 0.01 (− 0.05, 0.02)	0.361	− 0.04 (− 0.08, − 0.00)	0.046
FPG (mmol/L)	− 0.28 (− 0.43, − 0.12)	< 0.001	− 0.25 (− 0.60, 0.10)	0.168	− 0.31 (− 0.55, − 0.07)	0.013	− 0.26 (− 0.51, − 0.01)	0.038
TG (mmol/L)	0.30 (0.19, 0.40)	< 0.001	0.74 (0.46, 1.02)	< 0.001	0.30 (0.11, 0.49)	0.002	0.22 (0.08, 0.35)	0.002
TC (mmol/L)	0.31 (0.18, 0.45)	< 0.001	0.78 (0.43, 1.13)	< 0.001	0.39 (0.18, 0.61)	< 0.001	0.10 (− 0.11, 0.31)	0.341
LDL-C (mmol/L)	0.55 (0.37, 0.73)	< 0.001	0.81 (0.38, 1.25)	0.001	0.62 (0.33, 0.91)	< 0.001	0.42 (0.15, 0.68)	0.002
HDL-C (mmol/L)	− 0.11 (− 0.62, 0.41)	0.688	0.56 (− 0.54, 1.66)	0.323	− 0.04 (− 0.84, 0.75)	0.918	− 0.55 (− 1.40, 0.30)	0.206
ALT (U/L)	0.01 (0.01, 0.01)	< 0.001	0.01 (− 0.00, 0.01)	0.175	0.01 (0.00, 0.01)	0.006	0.01 (0.01, 0.01)	< 0.001
eGFR (mL/min/1.73 m^2^)	0.06 (0.05, 0.06)	< 0.001	0.05 (0.04, 0.06)	< 0.001	0.06 (0.05, 0.06)	< 0.001	0.06 (0.05, 0.06)	< 0.001
Hypertension								
No (%)	0		0		0		0	
Yes (%)	− 0.77 (− 1.04, − 0.50)	< 0.001	− 1.19 (− 1.89, − 0.48)	0.001	− 0.76 (− 1.18, − 0.34)	< 0.001	− 0.66 (− 1.07, − 0.26)	0.002
Smoking								
No (%)	0		0		0		0	
Yes (%)	− 0.17 (− 0.45, 0.11)	0.228	− 0.26 (− 1.02, 0.50)	0.501	0.06 (− 0.37, 0.49)	0.775	− 0.38 (− 0.79, 0.03)	0.070
Drinking								
No (%)	0		0		0		0	
Yes (%)	− 0.28 (− 0.56, 0.01)	0.061	0.31 (− 0.49, 1.11)	0.444	− 0.30 (− 0.75, 0.16)	0.202	− 0.40 (− 0.82, 0.01)	0.059
Treatment, n (%)								
No (%)	0		0		0		0	
Yes (%)	− 0.07 (− 0.37, 0.23)	0.668	0.26 (− 0.54, 1.06)	0.532	0.05 (− 0.43, 0.52)	0.847	− 0.26 (− 0.71, 0.18)	0.244

BMI, body mass index; DBP, diastolic blood pressure; SBP, systolic blood pressure; FPG: fasting plasma glucose; SUA, serum uric acid; SCr, serum creatinine; SUA/SCr, serum uric acid to serum creatinine ratio; TG, triglyceride; TC, total cholesterol; LDL-C, low density lipoprotein cholesterol; HDL-C: high density lipoprotein cholesterol; ALT, alanine aminotransferase; eGFR, estimated glomerular filtration rate. *P* < 0.05 is considered to be statistically significant.

### Nonlinear relationship between LDL-C and SUA/SCr

**Figure 1:**
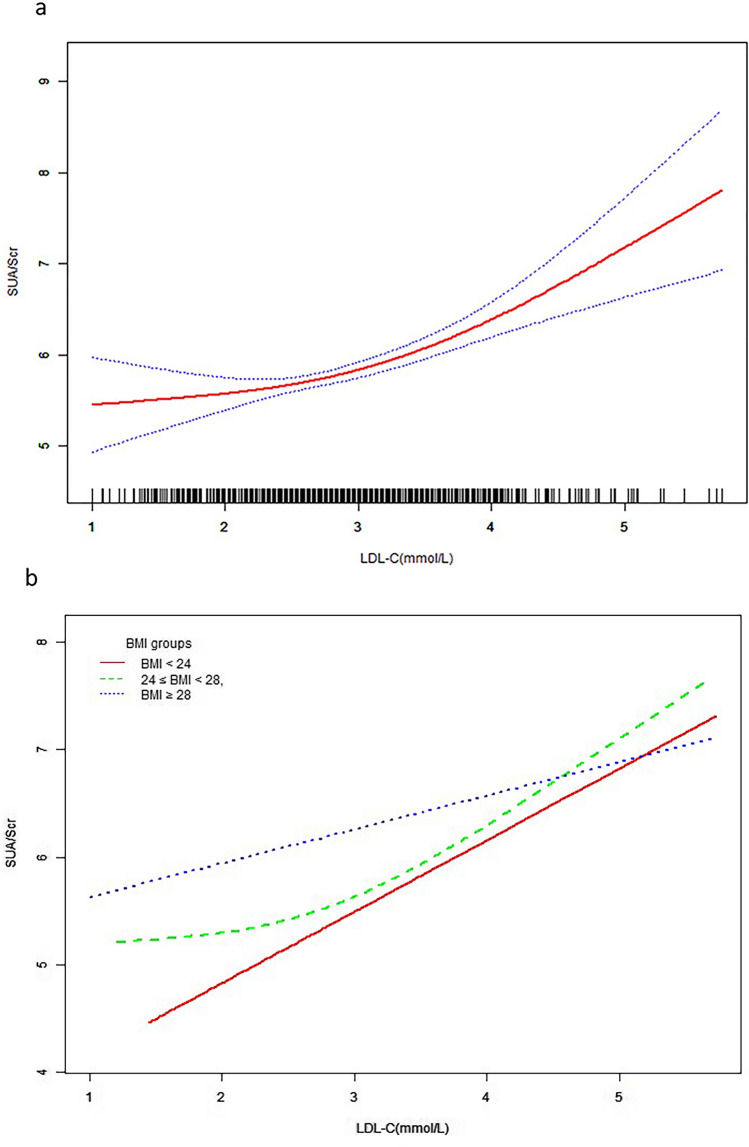
The relationship between LDL-C and SUA/SCr by smooth curve fitting. The curve fitting for LDL-C and SUA/SCr in all participants (**a**). The curve fitting for LDL-C and SUA/SCr stratified by BMI (**b**). Adjustment variables: age, BMI, drinking, hypertension, treatment, TG and ALT. LDL-C, low density lipoprotein cholesterol; SUA/SCr, serum uric acid to serum creatinine ratio; BMI, body mass index; TG, triglyceride; ALT, alanine aminotransferase.

Through smooth curve fitting, a nonlinear relationship was observed between LDL-C and SUA/SCr after adjustment for age, BMI, drinking, hypertension, treatment, TG and ALT. The fitted curve exhibited a turning point and two different trends. When LDL-C was over the turning point, there was a positive relationship between LDL-C and SUA; however, if LDL-C was below the turning point, no correlation was observed between them (Fig. 1.a).

### Effect of LDL-C levels on SUA/SCr under different BMIs

Furthermore, stratification analysis by BMI was performed in the studied population, after adjustment for age, BMI, drinking, hypertension, treatment, TG and ALT, the results showed that LDL-C was positively correlated with SUA/SCr in the group with a BMI of 24–28 (β = 0.51, 95%CI 0.22–0.80; *P* < 0.001) and BMI of 28 or greater (β = 0.30, 95%CI 0.05–0.55; *P* = 0.019), whereas no association was found in the group with BMI of less than 24 (*P* = 0.070, Table 3).In addition, we also conducted smooth curve fitting analysis in different BMI groups after adjusting for the above confounding factors. Interestingly, consistent with the overall population, we found a nonlinear relationship between LDL-C and SUA/SCr in men with a BMI of 24–28 (Fig. 1b). The multivariate piecewise linear regression model indicated that the inflection point of LDL-C was 2.6 mmol/L and 1.8 mmol/L in the overall population and in group with BMI of 24 to 28, respectively. When LDL-C was more than the turning points, SUA/SCr increased with increasing LDL-C levels (Table 4).

**Table 3:** The effect of LDL-C on SUA/SCr based on BMI stratification by multiple linear regression analysis.

Model	BMI < 24 (n = 119)		24 ≤ BMI < 28 (n = 421)		BMI ≥ 28 (n = 416)	
	β (95% CI)	*P* value	β (95% CI)	*P* value	β (95% CI)	*P* value
Model I	0.81 (0.38, 1.25)	< 0.001	0.62 (0.33, 0.91)	< 0.001	0.42 (0.15, 0.68)	0.002
Model II	0.82 (0.38, 1.25)	< 0.001	0.52 (0.24, 0.80)	< 0.001	0.34 (0.09, 0.58)	0.008
Model III	0.42 (− 0.03, 0.86)	0.070	0.51 (0.22, 0.80)	< 0.001	0.30 (0.05, 0.55)	0.019

Model I was unadjusted. Model II was adjust for age. Model III was adjust for age, BMI, drinking, hypertension, treatment, TG and ALT. LDL-C, low density lipoprotein cholesterol; SUA/SCr, serum uric acid to serum creatinine ratio; BMI, body mass index; TG, triglyceride; ALT, alanine aminotransferase. *P* < 0.05 is considered to be statistically significant.

**Table 4:** The threshold effect for the relationship between LDL-C and SUA/SCr.

Models	All		24 ≤ BMI < 28	
	β(95%CI)	*P* value	β(95%CI)	*P* value
Model I				
One line slope	0.43 (0.26, 0.60)	< 0.001	0.51 (0.22, 0.80)	< 0.001
Model II				
Turning point (K)	2.6		1.8	
< K slope 1	− 0.01 (− 0.48, 0.46)	0.963	− 2.82 (− 5.72, 0.09)	0.058
> K slope 2	0.61 (0.36, 0.86)	< 0.001	0.67 (0.35, 0.98)	< 0.001
LRT test	0.047		0.023	

Model I, linear analysis; Model II, non-linear analysis. LRT test, Logarithmic likelihood ratio test. (*P* < 0.05 means Model II is significantly different from Model I, which indicates a non-linear relationship); Adjustment variables: age, BMI, drinking, hypertension, treatment, TG and ALT. LDL-C, low density lipoprotein cholesterol; SUA/SCr, serum uric acid to serum creatinine ratio; BMI, body mass index; TG, triglyceride; ALT, alanine aminotransferase.

## Discussion

In our study, we observed that LDL-C levels were associated with SUA/SCr in male gout patients. More significantly, we found that the relationship between LDL-C levels and SUA/SCr was inconsistent across different BMIs. Through multivariate linear and piecewise regression analyses, we found a positive association in patients with a BMI of 28 or greater. Moreover, in patients with a BMI of 24–28, a nonlinear relationship between LDL-C and SUA/SCr was found, especially when the LDL-C levels were greater than 1.8 mmol/L, SUA/SCr increased with the increase of LDL, while LDL-C levels were lower than 1.8 mmol/L, no correlation was observed. However, we did not identify a correlation between LDL-C and SUA/SCr in patients with a BMI lower than 24. The results suggested that the relationship between LDL-C and SUA/SCr may be affected by BMI.

Urate is the final product of purine nucleotide metabolism and mainly excreted by the kidney, therefore, insufficient excretion of SUA due to renal disfunction can increase SUA concentrations, often, SUA levels are likely to increase when the eGFR is low. In previous studies, dyslipidaemia has been identified as a risk factor causing or contributing to kidney damage^27^, and it has been suggested that high LDL-C levels may impair arteria renalis and glomerular arterioles, leading to the development of atherosclerosis or occlusion, thus decreased SUA excretion occurs^28^. Consequently, if SUA is indeed associated with LDL-C, then renal function-normalization SUA, which may represent pure SUA production, would be a more accurate indicator of the relationship between LDL-C and SUA. In our study, we used biochemical analyzer to directly detect serum LDL⁃C, although Sampson and Friedewald formulas are convenient to use and cost effective, the Friedewald formula will underestimate LDL-C when TG levels are high or LDL⁃C levels are very low^29^, and the applicability of Sampson formula in Chinese population has not been evaluated. We found that LDL-C and TG were significantly associated with SUA/SCr in normal weight, overweight and obese groups, respectively. The correlation between TG and elevated SUA has been confirmed, and research suggests that TGs are lipolyzed into free fatty acids, which can activate purine synthesis and thereby increasing SUA^30^.As has been mentioned, the relationship between LDL-C and SUA/SCr showed a transition from nonlinear to linear as BMI increases, which suggested that BMI might play an important role in the relationship. However, Liang and colleagues found no association between LDL-C levels and SUA in patients with gout and asymptomatic hyperuricaemia, probably because most of the participants in their study were normal weight or overweight^31^.

Obesity is currently considered as a chronic low-grade inflammatory stress^32^, which is associated with hyperuricaemia, dyslipidaemia, insulin resistance, type 2 diabetes and hypertension. Based on our findings, we believe that overweight and obese gout patients are prone to metabolic disorders, making uric acid metabolism more vulnerable to other factors. Whether the positive correlation between BMI and SUA is modulated by LDL-C and the potential regulatory mechanism remain unclear. Indeed, previous studies have discovered that the LDL receptor binding levels in mononuclear cells were decreased in obese male subjects ^33^. When a weight loss of 10 kg occurred, the levels of LDL receptor binding increased significantly by 27.5%, and likewise, LDL-C levels decreased significantly^34^. Furthermore, a Mendelian randomization study observed a positive correlation trend between BMI and LDL-C (P > 0.05) both in observational and genetic estimates for LDL-C, and they demonstrated that the increased risk of obesity-induced ischaemic heart disease was mediated partly by elevated LDL-C levels^35^. Besides, a recent Mendelian randomization study showed that BMI has a causal effect on TGs but not on LDL-C, and TGs mediated a 14.1% effect of BMI on SUA^36^. Nevertheless, due to the different populations of the genetic association with LDL-C in the abovementioned MR studies, we believe that the relationship between BMI, LDL-C and SUA in different ethnic groups deserves further study.

The relationship between lipid profiles and SUA has shown controversial results. Studies from a National Health and Nutrition Examination Survey of Korea showed a more pronounced trend of correlation between SUA and LDL-C, TC and TG in male participants^37^. Conversely, a cross-sectional study from ethnic minorities in China demonstrated a U-shaped relationship between SUA and the risk of high LDL-C^38^. In the population of asymptomatic hyperuricemia of Qatar, they found that LDL-C and TG were statistically significantly higher than normouricemic group. However, when adjusting for age, gender, BMI, exercise and smoking, the associations were not statistically significant^39^. The differences in lipid metabolism may be related to sex-specific fat distribution^40,41^. Data from obese men after laparoscopic sleeve gastrectomy suggested that the improvement in SUA levels was correlated with the improvement in sex-specific fat distribution and elevated levels of total testosterone, and they believed that testosterone might play a role in that process ^42^. Furthermore, oestrogen is known to have a uricosuric effect ^43^, but there is a decreased synthesis of testosterone and oestrogen in male gout patients^44^, therefore, we speculated that sex hormones might play a role in the different relationships between lipid profiles and SUA. Further studies are needed to confirm this.

In addition, in our study, dyslipidaemia was found in 72.6% of the male gout patients, the high prevalence of dyslipidaemia indicated the necessity of lipid management in patients with gout, especially in overweight or obese men with gout. Based on our results, the finding of the LDL-C inflection points suggest that weight control and LDL-C reduction are equally important in the male gout population. Moreover, we found an inverse relationship between age and SUA/SCr, which suggests that SUA levels may decrease with increasing age, the result is consistent with those of recent studies in Koreans and Chinese^31,45^. In youth, men have a high risk of hyperuricemia or gout, which may be related to unhealthy living habits, such as meat-based diet, drinking, seafood, etc. These bad habits may be corrected with age, along with a decreasing trend in the incidence of hyperuricemia or gout^46^. However, another study showed that in postmenopausal women, the incidence rate of hyperuricemia increased with age^47^, which may be related to hormonal changes. Additionally, we found FPG was negatively associated with SUA/SCr, which was consistent with the results in Saudi type 2 diabetic patients^19^, the ultrafiltration state induced by hyperglycemia may promote renal excretion of SUA, which may partially explain the inverse correlation. Similarly, hypertension was also found to be inversely associated with SUA/Scr, perhaps due to the higher levels of creatinine in these hypertensive population. Besides, we found a positive correlation between ALT and SUA/Scr, while a positive correlation between ALT and SUA was also detected in patients with gout and asymptomatic hyperuricemia^31^.

Our study had some limitations. First, the design of the current cross-sectional study did not prove a cause-and-effect conclusion between lipid levels and hyperuricaemia. Second, the participants were limited to male patients with gout, and the results may not be applicable to female gout patients and gout patients complicated with diabetes. Third, although we have adjusted for some confounding factors, there may be confounders that have not yet been measured, such as diet, physical exercise, and education level. Therefore, it would be prudent to extend these conclusions, and additional large-scale prospective studies should be performed to explore the relationship between LDL-C and SUA/SCr in gout patients.

## Conclusions

In conclusion, dyslipidaemia is common in male gout patients. Using the data of Chinese male gout patients, we found a positive correlation between LDL-C levels and SUA/SCr in overweight and obese patients, which indicates that BMI may be involved in this relationship. Our study suggested that for overweight and obese male gout patients, attention should be given to the management of weight and LDL-C levels while taking uric acid lowering treatment. In the future, it may be necessary to set lipid management goals based on age, sex, BMI, comorbidities and other factors in gout patients.

## Data Availability

The datasets used and analyzed during the current study are available from the corresponding author on reasonable request.
